# Bio-Fabrication of ZnONPs from Alkalescent Nucleoside Antibiotic to Control Rice Blast: Impact on Pathogen (*Magnaporthe grisea*) and Host (Rice)

**DOI:** 10.3390/ijms24032778

**Published:** 2023-02-01

**Authors:** Taswar Ahsan, Bingxue Li, Yuanhua Wu, Zijing Li

**Affiliations:** 1 Department of Resources and Environmental Microbiology, College of Land and Environment, Shenyang Agricultural University, Shenyang 110866, China; 2 Department of Plant Pathology, College of Plant Protection, Shenyang Agricultural University, Shenyang 110866, China; 3 Food Science College, Shenyang Agricultural University, Shenyang 110866, China

**Keywords:** alkalescent nucleoside antibiotic, bio-fabrication, ZnONPs, *Magnaporthe grisea*, rice blast

## Abstract

In the traditional method of the bio-fabrication of zinc oxide nanoparticles (ZnONPs), bacterial strains face metal toxicity and antimicrobial action. In the current study, an alkalescent nucleoside antibiotic was mixed with zinc hexanitrate to fabricate the ZnONPs. An integrated approach of DIAION HP-20 macroporous resin and sephadex LH-20 column chromatography was adopted to separate and purify alkalescent nucleoside AN03 from *Streptomyces koyanogensis*. Alkalescent nucleoside was confirmed by the Doskochilova solvent system. The bio-fabricated ZnONPs were characterized by using Fourier transform infrared (FTIR), X-ray diffraction (XRD), and transmission electron microscopy (TEM) analyses. The XRD spectrum and the TEM images confirmed the crystallinity and the spherical shape of the ZnONPs with an average size of 22 nm. FTIR analysis showed the presence of functional groups, which confirmed the bio-fabrication of ZnONPs from alkalescent nucleoside ANO3. In-vitro studies showed that 75 μg/mL of ZnONPs had a strong inhibitory zone (28.39 mm) against the *Magnaporthe grisea* and significantly suppressed the spore germination. SEM and TEM observations respectively revealed that ZnONPs caused breakage in hyphae and could damage the cells of *M. grisea*. Greenhouse experiments revealed that the foliar spray of ZnONPs could control the rice blast disease by 98%. Results also revealed that ZnONPs had positive effects on the growth of the rice plant. The present study suggested that ZnONPs could be fabricated from microbe-derived nucleoside antibiotics without facing the problems of metal toxicity and antimicrobial action, thus overcoming the problem of pathogen resistance. This could be a potent biocontrol agent in rice blast disease management.

## 1. Introduction

Rice blast, a destructive disease that most rice-growing countries suffer from, is caused by *Magnaporthe grisea* [1]. Approximately 60% of the world’s population depends on rice for their diet. Rice is economically significant since it feeds 60% of the world’s population. By growing resistant varieties and adopting chemical approaches, blast infections could be efficiently managed [2]. However, genetically engineered plants and chemicals do not perform as stated in terms of efficacy and environmental safety [3]. A number of fungicides have been used to treat blast disease. However, synthetic fungicides, on the other hand, contaminate the environment, cause residue problems, contribute to pesticide resistance, damage soil health, and disrupt natural ecosystems [4]. Since bio-compounds are non-toxic to biota and the environment, they are becoming a major source of fungicide production [5]. Meanwhile, biocontrol agents face pathogen resistance [6] and have also been limited in their use in commercial agriculture due to inconsistencies in their performance. There are many biotic and abiotic factors that can affect biocontrol agents’ effectiveness. Depending on these conditions, their modes of action or multitrophic interactions may be altered. Likewise, a bacteria’s final levels of disease control are strongly influenced by pathosystem variables and environmental factors [7]. As a result, biological control agents (BCAs) and compatible protection additives are common, and they can be added at various stages of production. Traditional protective ingredients (sucrose, glycerol, and arabic gum) promote microbe survival, whereas adjuvants (surfactants, emulsifiers, dispersants, coupling agents, and stabilizing agents) make mixing, handling, application, and effectiveness easier [8]. The factors discussed above necessitate the immediate implementation of agricultural disease control measures [9].

Recent developments in nanotechnology may enable the control of agricultural pests in the near future [10]. Nanotechnology has resulted in the development of nanobiotechnology applications. These include nanoencapsulated pesticide delivery systems [11]. Recent decades have seen an increase in researchers’ interest in metals and their oxides due to their ability to withstand harsh conditions [12]. The stable, safe, and non-toxic properties of oxides of metals, such as zinc oxide, make them especially interesting [13]. Antifungal, antibacterial, antidiabetic, and acaricidal applications of zinc oxide nanoparticles have made them more attractive [14]. ZnONPs are also characterized by their ability to inhibit pathogenic fungi, largely due to the photo-oxidizing and photocatalytic properties they possess, as well as their ability to control infection in plants [15]. The synthesis of metallic nanoparticles can be carried out by various physicochemical methods, but many concerns exist, such as their cost, toxicity, complexity, and environmental impact [16]. The fabrication of nanoparticles (NPs) by green synthesis provides safe, environmentally friendly, and cost-effective alternatives to traditional approaches. By using microorganisms such as cyanobacteria, algae, plants, fungi, and lichens and naturally extracted biomolecules (i.e., pigments, vitamins, polysaccharides, proteins, and enzymes), bulk materials can be converted into nanoscale products [17]. Bacterial-mediated ZnONP production is relatively less prevalent [18]. Bacterial strains should also be non-pathogenic in order to adhere to the non-harmful notion of green chemistry [19]. The *Streptomyces* bacterium is a major source of several specialized metabolites that have medical and agricultural applications [20], as well as the capability of synthesizing a specific type of nucleoside [21]. Nucleoside antibiotics are a diverse group of natural compounds that serve several biological purposes [22,23]. In mammals and plants, nucleoside antibiotics are highly effective at fighting infections caused by pathogens [24]. This study takes into account the toxicity of conventional nanoparticles, as well as the fact that *Streptomyces* face resistance, lower efficiency, and stability issues in the environment. In order to achieve these goals, we adopted a reliable, efficient, sustainable, and environmentally friendly strategy. A *streptomyces* extracted nucleoside was used in the current study to fabricate ZnONPs. Bio-fabricated ZnONPs were also evaluated against the pathogen *Magnapothorte grisea* and its impact on host (rice) plants.

## 2. Results

**Figure 1:**
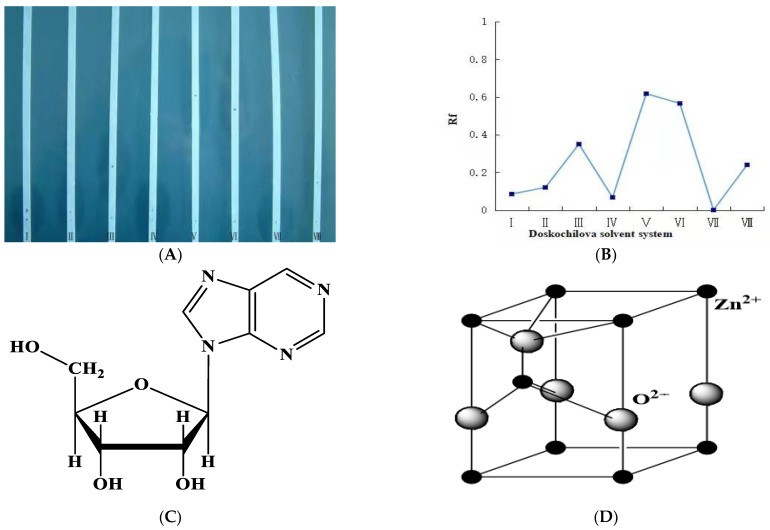
(**A**) Paper chromatogram of the effective component of AN03 in Doskochilova solution system. (**B**) Rf value of effective component of AN03 in the Doskochilova solvents system. (**C**) typical structure of alkalescent nucleoside antibiotic. (**D**) observed crystalline phase of ZnONPs indicates the typical hexagonal Wurtzite phase.

**Figure 2:**
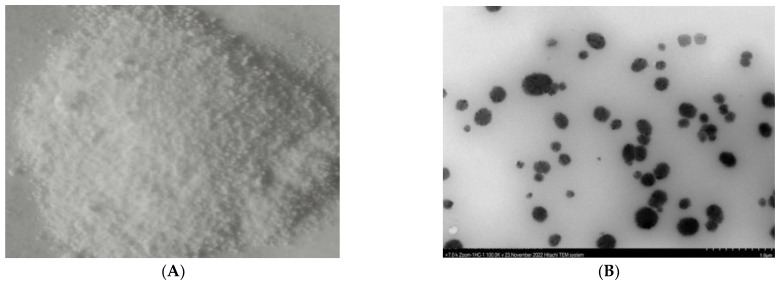
(**A**) bio-fabricated ZnONPs, whitish creamy substance. (**B**) TEM image of ZnONPs bio-fabricated by the alkalescent nucleoside antibiotic.

In the present study, we used a bio-method to synthesize the ZnONPs by using the nucleoside antibiotic ANO3 derived from the *Streptomyces Koyanogensis* TA-47 strain. Results showed that AN03 belongs to a water-soluble alkalescent nucleoside antibiotic and could be separated and purified by DIAION HP-20 macro reticular adsorbent and sephadex LH-20 gel filtration. The creamy whitish crude extract was obtained. The results of the Doskochilova solvent system confirmed that AN03 was a nucleoside antibiotic. The paper chromatograph is shown in Figure 1A. It appears that the active component in the isolated fractions had the highest Rf value in solvent system V, as well as in solvent system VI, which indicates that there were two active components, as shown in Figure 1B. An Rf value was calculated by dividing the distance traveled by the clear zone of an alkalescent nucleoside antibiotic by the distance traveled by the solvent front. The measurement was made after paper chromatography. It can be speculated that the isolated fractions contain nucleoside antibiotics that are alkaline and water-soluble. Figure 1C depicts a typical structure of nucleoside antibiotics. AN03 molecules were successfully capped by the zinc nitrate hexahydrate after reacting with it. A typical proposed structure of ZnONPs is shown in Figure 1D. After 24 h, a whitish precipitate was obtained, which indicated the reduction of zinc ions and the fabrication of ZnONPs, as shown in Figure 2A. Transmission electron microscope analysis showed that the ZnONPs are spherical in shape and slightly agglomerated. This indicated that AN03 capped well over Zn+, as shown in Figure 2B.

**Figure 3:**
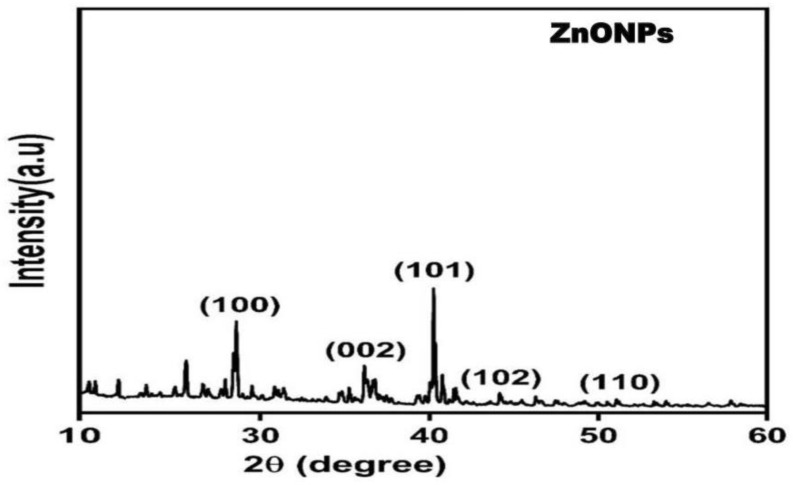
Patterns of bio-fabricated ZnONPs as measured by X-ray diffractometer.

An XRD analysis confirmed the composition and structure of biosynthesized ZnO nanoparticles. The ZnO nanoparticles displayed intense peaks on the XRD pattern, indicating high purity and crystalline characteristics (Figure 3). The peaks at 2θ = 29.50°, 36.50°, 40.50°, 45.60°, and 50.80°, were assigned to (100), (002), (101), (102), and (110), respectively. According to the results, oxide-specific peaks are clearly visible in the XRD pattern.

**Figure 4:**
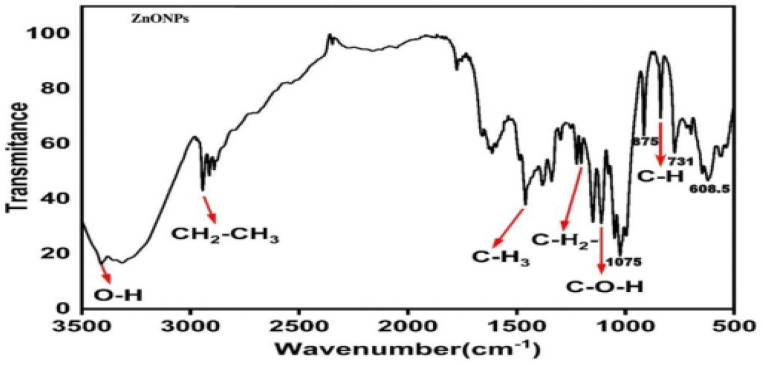
Fourier transform infrared spectra of bio-fabricated ZnONPs.

According to the Debye–Scherrer equation, ZnONPs’ average size corresponds to the maximum diffraction peak (101) and the average size was 22 nm. The analysis of Fourier transform infrared (Figure 4) showed six strong peaks for ZnONPs at 760, 1124, 1390, 1465, 2840, and 3400 cm^−1^. A strong and broad peak at 3400 cm^−1^ was related to alcoholic stretching O-H, and the vibration mode of the -OH group overlapped with stretching NH. The stretching C-H alkane group was represented by the medium peak at 2840 cm^−1^. The medium peak at 1465 cm^−1^ was caused by the bending alkane. Furthermore, a medium peak at 1390 cm^−1^ indicated aldehyde bending C-H. The high peak at 1124 cm^−1^ was caused by stretching the C-O-H secondary alcohol. The sharp peaks at 760 cm^−1^ were attributed to bending C-H benzene derivatives. The FT-IR study of ZnONPs revealed the participation of several groups in the reducing, capping, and stabilizing processes, including O-H, C-O-H, and C-H groups present in ANO3. The spectra correspond to the presence of functional groups of secondary alcohol, alkane, and amine regions. Results, also shown in Figure 4, show that the generation of the tetrahedral coordination of Zn causes the absorption at 875 cm^−1^. The C-O stretching vibration causes the bond at 1075 cm^−1^. Peaks in the range of 730 to 610 cm^−1^ represent the stretching vibrations of ZnONPs.

**Figure 5:**
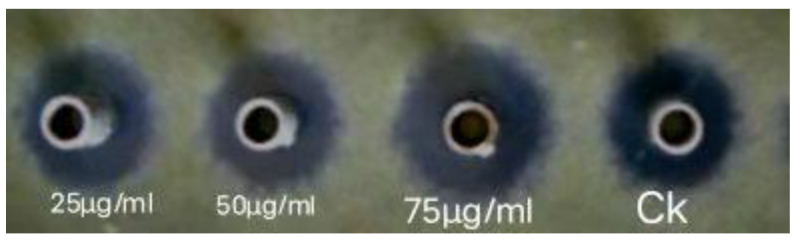
An illustration of antifungal activity of ZnONPs, against the *M. grisea* at different concentrations. In control (CK) treatment a commercial fungicides (Tebuconazole) with the dose of 100 μg/mL was used.

**Figure 6:**
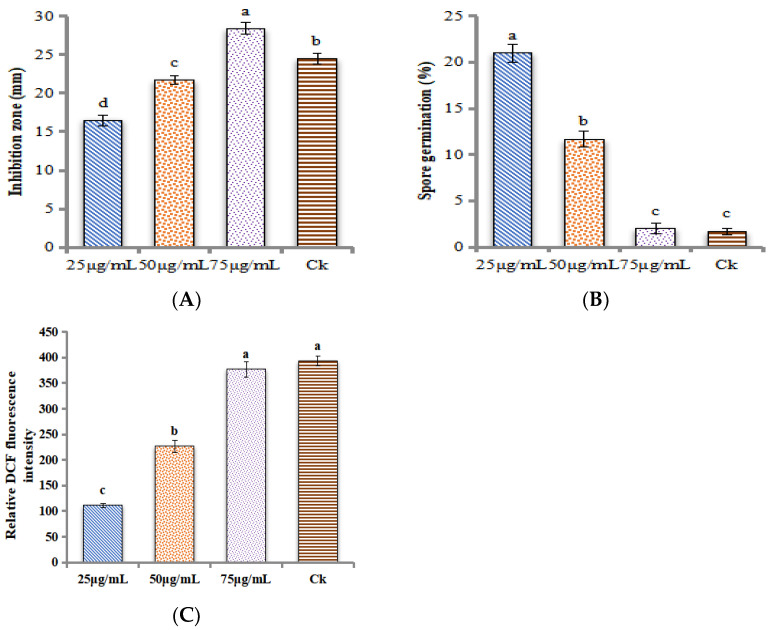
(**A**) Antifungal activity of ZnONPs, against the *M. grisea* at different concentrations of 25 μg/mL, 50 μg/mL, and 75 μg/mL ZnONPs. (**B**) The germination rates of spores of *M. grisea* treated with ZnONPs with the same concentration as in (**A**). (**C**) ROS level of *M. grisea* spore after incubation with the same concentrations of ZnONPs as in (**A**). In control (CK) treatments a commercial fungicides (Tebuconazole) with the dose of 100 μg/mL was used. Each value represents a comparison of the Mean ± SE. The letters a, b, c, and d indicate that they are statistically different when compared pairwise, according to Tukey’s HSD (*p* > 0.05). To analyze the data, each treatment was repeated three times.

In-vitro studies revealed that ZnONPs had potent antifungal activity, as shown in Figure 5. The concentration of 75 μg/mL of ZnONPs had a significant inhibition zone of (28.39 mm) against the *M. grisea* (Figure 6A). In-vitro studies revealed that the concentration of 75 μg/mL significantly controlled the spore germination, as only 2.00% of spores were germinated (Figure 6B). The control group (commercial fungicide) had a stronger control over spore germination, only 1.67% of spores germinated. A number of studies have revealed that ZnONPs are capable of inhibiting phytopathogenic fungi such as *Botrytis cinerea and Penicillium expansum* [25], *Fusarium oxysporum* [26], and *Erythricium salmonicolor* [27].

**Figure 7:**
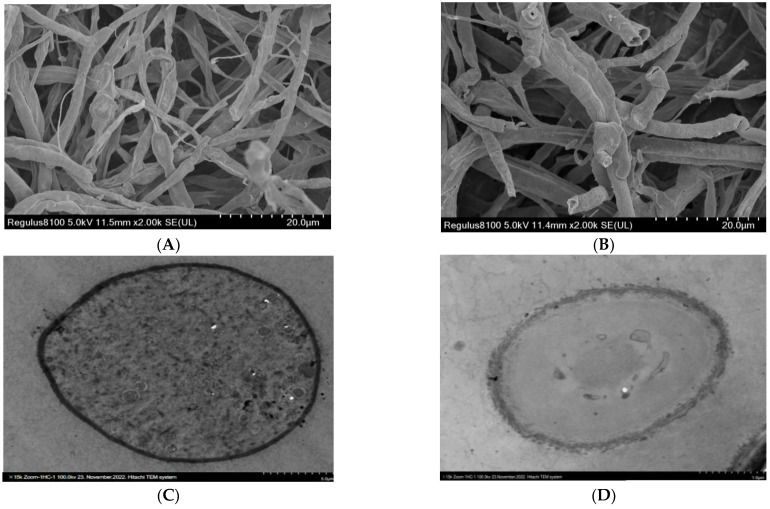
(**A**) SEM image of non-treated *M. grisea*. (**B**) SEM image of ZnONPs treated *M. grisea*. (**C**) TEM image of non-treated *M. grisea*. (**D**) TEM image of ZnONPs treated *M. grisea*.

Figure 6C showed that ZnONPs at the dose of 75μg/mL had a higher value of ROS generation in the *M. grisea*, as shown by 376.14 folds. The generation of ROS in fungal cells indicated the destruction of DNA and the rupturing of the cell membrane. Our results indicate that ZnONPs inactivate the oxidation–reduction balance by generating ROS, which may be associated with the mechanism of action of metallic NPs, such as pore formation in the cell membrane, and stipulating the transport of NPs into *M.grisea* cells. Scanning electron microscope analysis showed that ZnONPs significantly altered the morphology of the *M. grisea*. In Figure 7A, we show that the non-treated sample of *M.grisea* had a smooth structure and there was normal hyphae. However, as shown in Figure 7B, the ZnONPs-treated sample had broken hyphae, and its structural surface had become rough. Results in Figure 7C show that the non-treated sample of *M. grisea* had a normal shape and morphology with a very fine and clear cell structure. In Figure 7D, TEM analysis confirmed the destruction of the cell wall of *M. grisea* due to the effect of ZnONPs. It was clear from the results that the organelles of *M. grisea* were totally destroyed.

**Figure 8:**
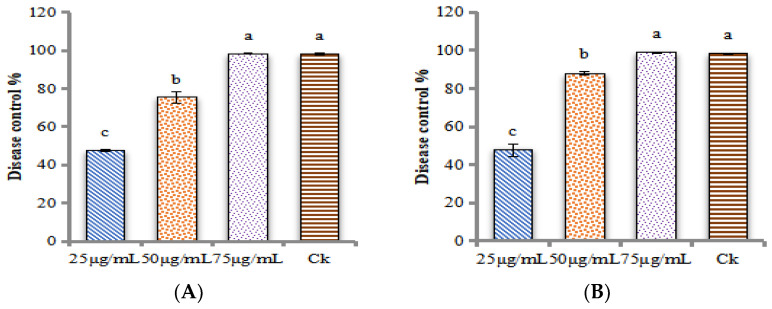
Disease control percentage of ZnONPs in green house conditions. (**A**) inoculation with *M. grisea* and foliar spray of ZnONPs (**B**) foliar spray of ZnONPs and inoculation with *M. grisea*. In control (CK) treatment a commercial fungicides (Tebuconazole) with the dose of 100 μg/mL was used. Different patterns of bar graphs representing different treatment levels. Each value represents a comparison of the Mean ± SE. The letters a, b, and c indicate that they are statistically different when compared pairwise, according to Tukey’s HSD (*p* > 0.05). To analyze the data, each treatment was repeated three times.

Figure 8A showed that inoculation of *M. grisea* and then foliar spraying of ZnONPs significantly controlled the disease. At the dose of 75 μg/mL of ZnONPs, the disease control efficiency was 98.39%. In the same way, in the opposite situation, foliar spraying of ZnONPs and then inoculation of *M. grisea* also showed significant disease control efficiency. At the dose of 75 μg/mL of ZnONPs, the disease control efficiency was 98.95%, as shown in Figure 8B. Results revealed that in the greenhouse experiment the foliar spraying of ZnONPs was more successful.

Table 1, demonstrate that ZnONPs had a significant influence on rice plant height and root length; specifically, when the plants were only treated with ZnONPs, the plant height and root length were 72.33 cm and 12.33 cm, respectively. Table 1 shows that the number of tillers on rice plants was significantly influenced by the ZnONPs. Specifically, when the plants were only treated with ZnONPs, the number of tillers increased by 5.33 n. Table 1, shows that the weight of a fresh plant was significantly influenced by the ZnONPs; when treated with ZnONPs, the weight of the plant increased to 99.9 g. Table 1 demonstrates that ZnONPs significantly influenced plant dry weight; specifically, when the plants were only treated with ZnONPs, the dry weight of the plants increased by 83.00 g. According to our investigation, in-vitro study proved that ZnONPs had significant antifungal potential against *M. grisea*, in comparison to the control (commercial fungicide). However, in greenhouse experiments, ZnONPs and the control had almost equal disease control efficiency.

## 3. Discussion

In the bio-method for the fabrication of NPs, microbe-produced compounds can be used either extracellularly or intracellularly [19]. According to our knowledge, this was the first investigation to fabricate the ZnONPs by using alkalescent nucleoside antibiotics derived from the *Streptomyces* strain. Usually, the bio-fabrication of NPs is carried out within the culture or during the fermentation process of the bacteria. Recently, a few studies reported that ZnONPs were prepared by these mechanisms, from these microbes: *Pseudochrobactrum* sp. [28], *Bacillus cereus* [29], *Paraclostridium* sp. [30], and *cyanobacteria*, such as *spirulina* [31]. Integrated chromatographic techniques could be an efficient, and cost-effective approach to extract the nucleoside. Recently, Sun [32], and Shirshekanb [33] was adopted this method to obtain macrocompounds. The results of the Doskochilova solvent system confirmed that AN03 was a nucleoside antibiotic. This chromatogram is similar to paper chromatograms of various antibiotics, including qingfengmycin, yunnanmycin, pyrimpeptidemycin, etc. [34]. AN03 molecules were successfully capped by the zinc nitrate hexahydrate after reacting with it. In this case, because NPs have high surface areas and have an agglomerative affinity with one another, they aggregate or agglomerate [35]. An XRD analysis confirmed the composition and structure of biosynthesized ZnO nanoparticles. Our results closely matched with the study of Barzinjy et al. [36], who synthesized the ZnONPs from the *Eucalyptus globulus Labill*. leaf extract with zinc nitrate hexahydrate. According to the results, oxide-specific peaks are clearly visible in the XRD pattern. These findings are highly compatible with those of the zinc nanoparticles synthesized by bacteria *Aeromonas hydrophila* by Jayaseelan et al. [37]. The reason for the small observed peaks at the various 2 theta values can be related to the crystallization of bacterial metabolites such as proteins and organic substances that coated the ZnONPs surface, as reported previously [38]. The spectra correspond to the presence of functional groups of secondary alcohol, alkane, and amine regions. These functional groups have a role in the stability/capping of nanoparticles. The presence of these different functional groups would mainly be due to biological synthesis [37,39]. Our results are very close to the ZnONPs produced from *Curcurm longa* tubers extracts reported by Javayarambabu et al. [40]. A number of studies have revealed that ZnONPs are capable of inhibiting phytopathogenic fungi such as *Botrytis cinerea* and *Penicillium expansum* [25], *Fusarium oxysporum* [26], and *Erythricium salmonicolor* [27]. Nanomaterials can generate the ROS, which leads to the deformation of the protein structures [41]. It was clear from the results that the hyphae of *M. grisea* were totally destroyed. In one previous study, ZnONPs showed the obvious destruction of the ultrastructure of the *Erythricium salmonicolor* fungus [27]. Foliar spraying of ZnONPs in pot experiments was found to be more effective than seed priming in increasing plant dry weight and controlling the effect of *Pectobacterium betavasculorum*, *Meloidogyne incognita*, and *R. solani*, the causal disease complex of beetroot (*Beta vulgaris* L.) [42]. Recent studies have revealed that ZnONPs significantly influence plant growth [43,44]. According to our investigation, in-vitro study proved that ZnONPs had significant antifungal potential against *M. grisea*, in comparison to the control (commercial fungicide). However, in greenhouse experiments, ZnONPs and the control had almost equal disease control efficiency.

## 4. Material and Methods

### 4.1. Microorganism

A pathogen and antagonist both were used in our initial work [6]. The *Streptomyces Koyanogensis* TA-47 strain was identified and screened against the *Magnaporthe grisea*. The bacterial strain was grown on nutrient agar media (NA) and stored at 4 °C, while *Magnaporte grisea* was grown on potato dextrose agar (PDA) and stored at 4 °C until the next experiment.

### 4.2. Separation, Purification, and Extraction of Alkalescent Nucleoside NA03 Antibiotic

We prepared the fermentation broth and centrifuged it as in our initial study [6]. An integrated approach of DIAION HP-20 macroporous resin and sephadex LH-20 column chromatography was adopted to separate and purify alkalescent nucleoside AN03. The crude extract of AN03 had been treated with oxalic acid before being added to the column. Among its characteristics, DIAION HP-20 has a pore volume of 1.3 mL, a specific surface area of 600 m^2^/g, a pore radius greater than 20 nm, an apparent density of 680 g/L-R, and a uniformity coefficient of 1.6. In this method, 5 mL of AN03 crude extracts are adjusted to pH2 with 1 mol/L oxalic acid, the sample loading speed is 0.5 mL/min, the desorption solution is 5–50% acetone, the speed is 0.25 mL/min, and the amount of desorption solution is about two times the column capacity. The acetone was removed by heating it in a water bath at 60 °C, and the biological activity of each tube was assessed using the Oxford cup method. DIAION HP-20 resins, and sephadex LH-20 column, both were purchased from sigma-aldrich, Shenghai, China.

Sephadex LH-20 column chromatography was used for the AN03 final extraction. To fully swell the sephadex LH-20 dry gel powder, we added it to a 70% methanol eluent and let it soak for 6 h. After that, we wet-packed the column and then equilibrated it by adding eluent that is roughly twice as large as the column volume, until the baseline was stable. We poured the sample onto the inner wall of the column using a pipette gun after passing it through a microporous membrane. Based on the polarity of the active component, we eluted the methanol at 0.1 mL/min in a gradient of 25–75%. In separate tubes, samples were collected at a rate of 2 mL/tube, and methanol was heated and volatilized at 60 °C. Antifungal activity was assessed using the Oxford cup method. For the AN03 crude extract, the combined active fraction was collected, precipitated with 100% ethanol repeatedly, and freeze-dried.

### 4.3. Doskochilova Solvent System for the Confirmation of Alkalescent Nucleoside AN03

The Doskochilova solvent system of [34] was used to confirm the type of alkalescent nucleoside AN03. The following eight solvent systems include: (I) water-saturated n-butanol; (II) water-saturated n-butanol containing 2% p-toluenesulfonic acid; (III) butanol: acetic acid: water (2:1:1); (IV) water-saturated n-butanol containing 2% hexahydropyridine; (V) 0.5 mol/L pH7.0 phosphate buffer saturated with n-butanol; (VI) water saturated with n-butanol, containing 2% p-toluenesulfonic acid; (VII) benzene: methanol (4:1), where the filter paper was treated with 0.5 mol/L pH7.0 phosphate buffer; and (VIII) 75% methanol, 25% water (containing 3% sodium chloride), where the filter paper was treated with 5% sodium sulfate. The sampling method is the same as that of pH chromatography, and the sampling volume is 15 µL. A clean bench was used to dry the filter paper strips after layering, and for 30 min, the strips were sterilized with ultraviolet light. The biological imaging method was adopted to determine the retardation factor (Rf). The Rf value was calculated by dividing the distance traveled by the clear zone of an alkalescent nucleoside antibiotic by the distance traveled by the solvent front. The measurement was made after paper chromatography.

### 4.4. Bio-Fabrication of ZnONPs

The fabrication of ZnONPs was carried out by adopting the method of Barzinjy et al., [36] with small variations. *Streptomyces Koyanogensis*-derived nucleoside antibiotic ANO3 was used for the bio-fabrication of ZnONPs. The extracted paste of AN03, which amounted to about 15 g, was added to the beaker and progressively warmed. Later on, when the temperature reached 60 °C, 3 g of zinc nitrate hexahydrate was mixed with the ANO3. The mixture was continually swirled for an hour at a temperature of 60 °C until it turned into a whitish paste. In order to achieve NPs with the highest yield, the temperature of the reaction had to be 60 °C. Afterward, the paste was blazed in a furnace at 400 °C for about 2 h, and then the residual was washed with ethanol and distilled water several times. The powder was then heated at 100 °C to dry. Then zinc oxide nanoparticles were obtained and they were ready for characterization.

### 4.5. Characterization of ZnONPs

To determine the phase purity and crystallinity of the structure of ZnONPs, they were determined by X-ray diffraction (XRD Modelle—D8 Advance, BRUKER, Germany). The size of nanoparticles is calculated by the below formula (Scherer Equation (1)):

D = kλ/βcosθ(1)

where D = average crystalline size perpendicular to the reflecting planes, K = shape factor, λ = X-ray wavelength, β = full width at the half maximum (FWHM), θ = diffraction angle.

Fourier transform infrared spectroscopy was used to analyze the functional group of nanoparticles (FTIR-jascov-650 spectrophotometer, Tokyo, Japan). ZnONPs were observed under a transmission electron microscope (TEM, Hitech model s-3400 n, Tokyo, Japan).

### 4.6. In-Vitro Experiments

#### In-Vitro Antagonistic Assay of ZnONPs against *M. grisea*

The Oxford cup method of Vincent et al. [45] was used to assess the antagonistic assay of ZnONPs against *M. grisea*. The following formulation was used to analyze the antifungal activity, spore germination, and reactive oxygen species (ROS): ZnONPs (25 μg/mL, 50 μg/mL, and 75 μg/mL), whereas a commercial fungicide, tebuconazole, at a dose of 100 μg/mL, was used as a control. PDA was poured into the petri dishes, and the spore suspension of *M. grisea* was mixed into it. Later on, we added 200 μL of the above-mentioned concentration of each treatment. After that, the plates were placed in an incubator for 14 days at 28℃. To determine the antifungal activity zone, the inhibition of *M. grisea* was measured. A further method for each parameter is as follows:

### 4.7. Evaluating Morphological Alterations in Fungal Mycelia Using Scanning Electron Microscopy (SEM) and Transmission Electron Microscopy (TEM)

We used scanning electron microscopy (SEM) to investigate changes in the morphology and ultrastructure of fungal hyphae after treatment with ZnONPs. NP-treated and control *M. grisea* mycelium were removed and fixed with glutaraldehyde (2%) at 4 °C for 4 h. The fixed sample was then rinsed several times with phosphate-buffered saline (PBS pH 7, 0.5 M) following the fixation with aqueous-osmium-tetroxide (1%). Then, a gradient series of ethanol (at concentrations of 30: 50: 70: 80: 90: and 100%) was used to exsiccate the hyphal samples for 20 min. The hyphal portions were then immersed in isoamyl acetate for an entire night. The segments were then coated with conductive gold sputter, subjected to an analytical amount of dry CO_2_, and examined under a SEM and TEM (S-4800, Hitachi, Japan).

### 4.8. Reactive Oxygen Species (ROS) Assay

To determine the reactive oxygen species (ROS) production in fungal spores induced by nanomaterials, an ROS assay kit was used, which contained the non-fluorescent molecule 2′, 7′-dichlorodihydrofluorescein diacetate (DCFH-DA). After being exposed to 200 µL of each concentration (25, 50, and 75 µg/mL) of ZnONPs for 2 h at 30 °C, spore cells (105 per mL) were rinsed three times with 0.1 M PBS (PH 7.8) and then recovered in PBS solution. The spores were given sterilized water as a positive control. The cell suspension received two liters of DCFH-DA (30 M), and it was grown for an additional hour in total darkness. An (Edinburgh FLS920 spectrometer, Livingston, UK) was used to detect the fluorescence intensity at 522 nm. Fluorescence intensity was compared between a sample and a control to determine intracellular ROS levels [28].

### 4.9. Green House Experiments

#### 4.9.1. Growth Conditions

In greenhouse settings, ZnONPs were tested for their ability to suppress *M. grisea*, which controls rice blast, and their impact on rice plants. The rice variety 1121 “white basmati rice” (*Oryza sativa* L.) was chosen as the host plant because it is particularly vulnerable to rice blast disease. All the experiments were performed in the greenhouse under the same control conditions. The paddy field was set up inside the greenhouse. Treatments were given to the plant after 45 days of being seedling transplanted in the paddy soil. There was no chemical treatment of the paddy soil or rice plants.

#### 4.9.2. Disease Control Effects of ZnONPs against the *M. grisea*

To study the disease effects, the formulation was set up as follows: ZnONPs (25 mg/L, 50 mg/L, and 75 mg/L), while the control group (CK) was treated with commercial fungicide Tebuconazole at the dose of 100 μg/mL per plant. The experimental approach was adopted in following way: (a) ZnONPs spray pre-inoculums of *M. grisea* (b) ZnONPs spray post-inoculums of *M. grisea*. The same method was followed for the control (CK). Disease control efficacy was determined by infection lesions on plants. All the treatments were replicated three times to analyze the data.

#### 4.9.3. Treatments to Determine the Impact of ZnONPs on Rice

The effects of ZnONPs were determined in the greenhouse conditions mentioned above, with and without the biotic stress of *M. grisea*. To evaluate the impact of ZnONPs on rice, the following treatment approaches were adopted: (a) Inoculated with M. grisea; (b) Spray of ZnONPs + inoculated with M. grisea; (c) Spray of ZnONPs; (d) control, no treatment was given. The dose concentration of ZnONPs for all affected plants was 75 mg/mL. All the treatments were given in triplicate. After the treatments, the following different parameters were evaluated according to particular methods:

#### 4.9.4. Effects of ZnONPs on Rice Plant Growth Parameters

After the 21 days of above-mentioned treatments, random plants were selected to study plant height, root length, number of tillers, fresh weight of plant, and dry weight of plant. All the experiments were performed in triplicate.

### 4.10. Statistical Analysis

The data were analyzed using one-way analysis of variance (ANOVA), and Tukey’s HSD was used to test for significance at *p* > 0.05. The statistical analysis was performed by using software IBM-SPSS version 25.0. New York, NY, USA.

## 5. Conclusions

In the current study, bioactive nucleoside antibiotic ANO3 was extracted from fermentation broth produced by *Streptomyces Koyanogensis* TA-47 and mixed with zinc hexanitrate to fabricate the ZnONPs. An integrated approach of DIAION HP-20 macroporous resin and sephadex LH-20 column chromatography was adopted to separate and purify the ANO3. The Doskochilova solvent system confirmed that ANO3 belongs to nucleoside antibiotics. The results of XRD spectrum and the TEM images revealed the crystallinity and the spherical shape of the ZnONPs. FTIR analysis showed the presence of functional groups, which confirmed the bio-fabrication of ZnONPs from ANO3. In-vitro studies showed that ZnONPs had strong antifungal activity against the *M. grisea* and significantly suppressed the spore germination. SEM and TEM analysis indicated that ZnONPs damaged the *M. grisea*. Further ROS assay analysis confirmed the cell burst of *M. grisea* due to the effect of ZnONPs. Greenhouse experiments revealed that the foliar spray of ZnONPs could control the rice blast disease. Results also revealed that ZnONPs had positive effects on the growth of rice plant. This could be a potent biocontrol agent in rice blast disease management.

## Figures and Tables

**Table 1:** Impact of ZnONPs on the growth of rice plant.

Treatment			Mean ± SE		
	Plant Height (cm)	Root Length (cm)	Number of Tillers (n)	Weight of Fresh Plant (g)	Weight of Dry Plants (g)
Inoculated with *M. grisea*	42.33 ± 1.33 d	4.33 ± 0.33 c	3.00 ± 0.58 b	48.33 ± 3.33 c	37.17 ± 1.59 c
ZnONPs + Inoculated with *M. grisea*	63.00 ± 0.58 b	7.33 ± 0.88 b	4.00 ± 0.58 ab	63.00 ± 3.51 b	48.57 ± 4.30 b
ZnONPs	72.33 ± 2.40 a	12.33 ± 0.33 a	5.33 ± 0.67 a	99.00 ± 5.86 a	83.00 ± 3.51 a
CK	55.00 ± 2.08 c	8.00 ± 0.58 b	4.00 ± 0.58 ab	64.33 ± 2.91 b	39.00 ± 3.21 bc

Means sharing similar letter in a column are statistically non-significant (*p* > 0.05). CK is the control group without the treatment.

## Data Availability

The data presented in this study are available on request from the corresponding authors.
